# Inclusion of butterfly pea hay in corn silage (with or without cobs) increases nutrient intake and carcass weight in lambs

**DOI:** 10.1007/s11250-026-05361-7

**Published:** 2026-09-29

**Authors:** Bruno Augusto de Souza Almeida, Fabio Andrade Teixeira, Timóteo Silva dos Santos Nunes, Glayciane Costa Gois, Salete Alves de Moraes, Lucas Oliveira Reis, Ery Jonhons Nascimento Ramos, Arquinor Conceição Rodrigues, Daniel Ribeiro Menezes, Elisvaldo José Silva Alencar, Adeilson de Melo Silva, Rafael Torres de Souza Rodrigues, Tiago Bresolin, Mário Adriano Ávila Queiroz

**Affiliations:** 1 Universidade Estadual do Sudoeste da Bahia –Itapetinga, Itapetinga, BA Brazil; 2 Universidade Federal do Vale do São Francisco - Petrolina, Petrolina, PE Brazil; 3 Universidade Federal do Recôncavo da Bahia - Centro de Ciências Agrárias, Ambientais e Biológicas, Cruz das Almas, Bahia Brazil; 4 Empresa Brasileira de Pesquisa Agropecuária, Petrolina, PE Brazil; 5 Department of Animal Sciences, University of Illinois at Urbana- Champaign, Urbana, IL USA

**Keywords:** Crude protein, Forage conservation, Intake, Starch, Weight gain

## Abstract

This study evaluated the effects of mixed corn silages, with and without commercially viable cobs, combined with butterfly pea (*Clitoria ternatea L.*) hay on intake, digestibility, water balance, nitrogen balance, and carcass characteristics of lambs. Twenty crossbred intact male lambs (18.0 ± 4.8 kg body weight) were distributed in a randomized complete block design with a 2 × 2 factorial arrangement and five replicates per treatment. Treatments consisted of corn silage with cobs (CSC), CSC plus butterfly pea hay (BH), corn silage without cobs (CSWC), and CSWC plus BH. Butterfly pea hay inclusion increased dry matter intake (*P* < 0.001), crude protein digestibility (*P* = 0.006), nitrogen absorption (*P* = 0.006), hot carcass weight (*P* = 0.011), and cold carcass weight (*P* = 0.009). Corn cobs improved starch digestibility (*P* = 0.015), water balance (*P* = 0.021), and hot carcass yield (*P* = 0.021). Average daily gain was not significantly affected, although a numerical increase was observed in lambs fed diets containing BH (*P* = 0.062). The inclusion of butterfly pea hay improved nutrient utilization and carcass weight, while the maintenance of corn cobs enhanced starch utilization and water balance in lambs fed corn silage-based diets.

## Introduction

Maize (*Zea mays L*.) is a key crop in animal feeding systems, particularly for confined small ruminants, due to its high nutritional value and widespread market availability (Diógenes et al., 2023). Its suitability for silage production stems from its high soluble carbohydrate content, low buffering capacity, and favorable microbial flora (Almeida et al. 2025). Maize silage meets most nutritional requirements of livestock and can reduce concentrate supplementation by up to 50% (Gülümser et al. 2021). However, variations in plant composition can alter its ensiling properties, negatively affecting silage quality (Souza et al. 2022).

A common practice in silage production is to partially or completely remove green cobs for commercial sale, leaving only residual plant material (Paula et al. 2021). This practice, combined with the low dry matter and soluble carbohydrate content of the remaining biomass, may compromise silage quality (Souza et al. 2022), leading to dry matter losses and undesirable organic acid formation. In addition, cob removal reduces the starch concentration and energy density of maize silage, which may negatively affect nutrient utilization and animal performance (Souza et al. 2022; Hilscher et al. 2019; Almeida et al. 2025). Additionally, maize silage typically has a low protein content (6.5–8.5%; Hilscher et al. 2019), limiting its use in high-demand diets (Gülümser et al. 2021). Incorporating other forage species to produce mixed silages could enhance both nutritional and fermentative characteristics.

The low protein content of maize silage can be addressed by incorporating legumes, which can increase its protein concentration (Hawu et al. 2022). However, producing mixed silages with maize and legumes requires synchronized harvesting to ensure both crops are at their ideal ensiling stage. Consequently, mixed silages of maize (with or without cobs) and a forage legume may offer a viable alternative to reduce protein concentrate costs while maintaining a balanced diet for optimal animal performance (Meng et al. 2022).

Butterfly pea (*Clitoria ternatea L.*) is a leguminous plant widely distributed across tropical, subtropical, and semi-arid regions due to its adaptability and resistance to climatic variations (Lemos et al. 2021). Butterfly pea has a dry matter content of 28.6–29.1% (Araújo et al. 2022), crude protein content ranging from 14% to 27%, dry matter digestibility of approximately 70% (Umami et al. 2022), and neutral detergent fiber content of 60.4% (Araújo et al. 2022). Previous studies with small ruminants have demonstrated that butterfly pea may improve nutrient intake, digestibility, and animal performance when included in forage-based diets (Araújo et al. 2022; Lemos et al. 2021). Its nutritional profile can significantly improve the characteristics of maize silage when used in mixed silages, promoting balanced diets for small ruminants. Additionally, butterfly pea acts as an absorbent additive when combined with maize during ensiling, reducing nutrient losses through leaching (Macêdo et al. 2021).

To date, no studies have examined the effects of maize cob inclusion combined with butterfly pea hay in mixed silages on lamb performance. We hypothesized that the inclusion of butterfly pea hay would improve the nutritional quality of maize silages and mitigate the nutritional limitations associated with cob removal, thereby improving nutrient intake, digestibility, nitrogen utilization, and carcass characteristics of lambs. Therefore, the objective of this study was to evaluate intake, nutrient digestibility, water balance, nitrogen balance, and carcass characteristics of lambs fed mixed maize silages with and without commercially viable cobs combined with butterfly pea hay.

## Materials and methods

### Study location and production of silages

The experiment was conducted at the Agricultural Sciences Campus of the Universidade Federal do Vale do São Francisco (UNIVASF) in Petrolina, Pernambuco, Brazil (9°23’55” S, 40°30’03” W; 376 m altitude).

The AG-1051 corn hybrid used for silage production came from an experimental area of approximately 3533 m^2^ and was harvested 75 days after planting, before 2/3 of the milk line. Butterfly pea was harvested – leaves, branches, petioles, and pods were manually cut 15 cm above the ground – from an experimental area of approximately 2400 m^2^ 110 days after planting, when the plants were in the flowering stage. Butterfly pea haymaking was carried out in the field, where all the material was dried for 72 h until it reached the hay point (91.60% dry matter), collected, and stored in a dry place.

All material was processed in a stationary forage harvester (Nogueira Pecus 9004, Saltinho – SP, Brazil) to an average particle size of 10–25 mm for the corn and 5–10 mm for the butterfly pea hay. Samples of the chopped material were collected for subsequent chemical analysis (Table 1). The mixed corn and butterfly pea silage consisted of 70% corn—with or without ears—and 30% butterfly pea hay (BH), on a fresh-weight basis. The inclusion level of 30% butterfly pea hay was selected based on previous studies demonstrating improved fermentative stability and nutritional quality of mixed silages at this proportion (Almeida et al. 2025), while maintaining adequate dry matter content for proper ensiling. The silages were produced in silage bags (120 × 52 cm, 140 μm thick, and 35 kg capacity). The ensiling process was carried out using a silage bagging machine (Laboremus SBL-35 N, 3 HP single-phase motor, Campina Grande – PB, Brazil). The corn and legume were manually homogenized according to the treatments prior to ensiling. The silos were compacted to an average density of 550 kg m⁻³ and stored at ambient temperature, averaging 27 °C. A sufficient number of silage bags was produced for each treatment to supply the animals throughout the entire experimental period. After sealing, the silos were weighed and re-weighed 60 days later, at the time of opening. To determine the fermentative and nutritional composition of the silages (Table 2), samples were collected upon opening the silos, after discarding the initial 10 cm layer.

**Table 1:** Chemical composition of ingredients in experimental diets

Items (in % dry matter)	Corn with cobs	Corn without cobs	Butterfly pea hay	Ground grain corn	Cottonseed bran	Urea
Dry matter*	22.23	21.84	91.60	89.95	91.00	97.50
Mineral matter	5.49	5.12	6.95	2.20	5.00	0.20
Organic matter	94.51	94.88	93.05	97.80	95.00	99.80
Crude protein	8.96	8.83	13.20	8.10	29.00	280.0
Ether extract	1.63	1.62	2.43	3.50	7.00	-
NDFap	64.19	60.22	41.23	10.8	40.00	-
Acid detergent fiber	30.73	29.93	29.28	3.55	36.00	-
Hemicellulose	33.45	30.29	11.95	7.25	4.00	-
Cellulose	16.08	15.97	4.10	3.80	23.58	-
Lignin	10.30	10.54	22.58	0.10	8.25	-
Starch	26.80	14.33	12.25	65.00	1.00	-
Total carbohydrates	83.92	84.43	77.42	86.20	59.00	-
NFC	19.73	24.21	36.19	75.40	19.00	-
TDN	56.35	54.24	59.63	80.25	57.41	-
NDIN	0.59	0.62	0.34	3.30	-	-
ADIN	0.12	0.16	0.25	1.59	-	-
NDIP	3.70	3.87	2.14	-	6.80	-
ADIP	0.73	1.02	1.59	-	6.55	-

*in % natural matter; NDFap: Neutral detergent fiber corrected for ash and protein; NFC: Non-fibrous carbohydrates; TDN: Total digestible nutrients; NDIN: Neutral detergent insoluble nitrogen; ADIN: Nitrogen insoluble in acid detergent; NDIP: Neutral detergent insoluble protein; ADIP: Acid detergent insoluble protein

**Table 2:** Nutritional composition, pH, and organic acids of mixed ensiled corn with cobs + butterfly pea hay silages (CSC + BH); ensiled corn without cobs + butterfly pea hay (CSWC + BH), ensiled corn with cobs without butterfly pea hay (CSC); and ensiled corn without cobs without butterfly pea hay (CSWC) were used in lamb fed

Items (g/kg dry matter)	Silages			
	CSC + BH	CSWC + BH	CSC	CSWC
Dry matter (g/kg natural matter)	410.8	403.9	210.5	208.6
Organic matter (g/kg dry matter)	936.4	930.2	953.4	946.4
Crude protein (g/kg dry matter)	110.5	109.2	96.4	100.1
Ether extract (g/kg dry matter)	21.4	14.3	17.7	16.2
Neutral detergent fiber (g/kg dry matter)	509.7	474.1	628.8	575.2
Acid detergent fiber (g/kg dry matter)	306.1	287.0	302.8	290.8
Total digestible nutrients (g/kg dry matter)	625.3	640.1	575.5	597.9
Hemicellulose (g/kg dry matter)	206.6	187.0	327.3	288.1
Cellulose (g/kg dry matter)	75.1	56.1	140.0	139.2
Lignin (g/kg dry matter)	183.9	195.1	100.9	102.8
Total carbohydrates (g/kg dry matter)	804.7	806.6	841.7	829.9
Non-fibrous carbohydrates (g/kg dry matter)	333.1	368.0	246.4	280.5
pH	3.26	3.20	2.94	2.94
Acetic acid (g/kg dry matter)	10.4	9.9	9.2	8.4
Propionic acid (g/kg dry matter)	0.7	0.8	1.2	0.8
Butyric acid (g/kg dry matter)	5.5	3.8	4.1	4.0
Lactic acid (g/kg dry matter)	55.6	61.5	62.1	55.4

### Experimental design

Twenty crossbred intact male lambs (3 months of age, 18.0 ± 4.8 kg initial body weight) were allocated to individual pens (1.10 × 2.00 m) equipped with feeders and drinkers. The experiment lasted 94 days and was preceded by 20 days of adaptation of the animals to the experimental diets. At the beginning of the adaptation period, animals were identified, weighed, treated against endo and ectoparasites, and randomly allocated to the pens according to the treatments. The pens were allocated in a shed covered with zinc tile, with a ceiling height of 3.5 m and a dirt floor.

This was a randomized complete block experimental design, with initial weight as the blocking factor. The treatments were arranged in a factorial design of 2 (presence or absence of cobs in the silages) × 2 (inclusion or not of butterfly pea hay in the silages), with 5 replications. The experimental diets consisted of four silages ((1- ensiled corn with cobs without butterfly pea hay (CSC); 2- ensiled corn with cobs + butterfly pea hay (CSC + BH); 3- ensiled corn without cobs without butterfly pea hay (CSWC); 4- ensiled corn without cobs + butterfly pea hay (CSWC + BH), and concentrate based on ground corn grain, cottonseed meal, urea, and mineral supplement (Table 3). Cottonseed meal was added to diets without butterfly pea hay to meet crude protein requirements. The diets were formulated with a forage-to-concentrate ratio of 75:25 on an as-fed basis, formulated in accordance with the National Research Council (NRC 2007) recommendations for daily gains of 150 g.

**Table 3:** Percentage and chemical composition of experimental diets

Ingredients (in % dry matter)	Diets			
	CSC + BH	CSWC + BH	CSC	CSWC
Ground corn	20.73	20.73	9.91	9.91
Silages	75.00	75.00	75.00	75.00
Cottonseed bran	0.00	0.00	10.73	10.73
Mineral premix*	3.00	3.00	3.00	3.00
Urea	1.27	1.27	1.36	1.36
TOTAL	100.00	100.00	100.00	100.00
	Chemical composition (% DM)			
Dry matter**	44.01	42.36	30.38	29.39
Organic matter	92.73	92.75	93.82	93.69
Crude Protein	15.42	15.02	15.41	15.04
Ether extract	2.23	2.62	2.86	2.68
NDFap	40.79	44.13	51.26	54.15
Acid detergent fiber	21.04	25.26	20.17	27.27
Hemicellulose	19.74	18.87	31.09	29.88
Cellulose	3.53	2.80	8.54	9.13
Lignin	18.40	18.03	10.14	10.98
Starch	32.76	19.80	20.31	18.10
Total carbohydrates	75.08	75.11	75.55	75.97
Non-fibrous carbohydrates	34.29	30.98	24.29	21.82
Total digestible nutrients	60.88	59.93	60.94	58.76
NDIN	0.38	0.52	0.15	0.25
ADIN	0.23	0.21	0.21	0.15
NDIP	2.35	3.27	1.11	1.54
ADIP	1.45	1.30	1.33	1.01
Metabolizable Energy (Mcal/day)	2.65	2.65	2.55	2.55

Ensiled corn with cobs + butterfly pea hay (CSC + BH); ensiled corn without cobs + butterfly pea hay (CSWC + BH), ensiled corn with cobs without butterfly pea hay (CSC); ensiled corn without cobs without butterfly pea hay (CSWC). *Composition: 7.5% P; 19% Ca; 1% Mg; 7% S; 14.3% Na; 21.8% Cl; 500 ppm Fe; 300 ppm Cu; 4600 ppm Zn; 1100 ppm Mn; 80 ppm I; 405 ppm Co; 30 ppm Se; ** in % natural matter; NDFap: Neutral detergent fiber corrected for ash and protein; NDIN: Neutral detergent insoluble nitrogen; ADIN: Nitrogen insoluble in acid detergent; NDIP: Neutral detergent insoluble protein; ADIP: Acid detergent insoluble protein

### Intake and digestibility

Food was provided at 09:00 h and 16:00 h, and water ad libitum. The amount of food offered was calculated based on the previous day’s intake, with leftovers limited to no more than 10% of the offered quantity. Samples of food offered, and leftovers were collected weekly for chemical analysis. Daily dry matter intake (DMI) was obtained by the difference between the total DM of the consumed diet and the total DM present in leftovers. Nutrient intake was determined as the difference between the total nutrients in the consumed diet and those in the leftovers, based on total DM.

The digestibility trial was conducted after the 20-day diet adaptation period and comprised 5 days of adaptation to the metabolic cages followed by 5 days of total collection. The same animals used in the performance trial were distributed into metabolism cages equipped with feeders and waterers. Fecal collection bags were attached to the animals using adjustable harnesses positioned around the hindquarters to allow for complete fecal collection without restricting animal movement. Bags were emptied twice daily, and feces were weighed. A 10% subsample of the total feces from each animal was collected for further analysis. Samples were stored in a freezer at -20 °C.

### Water balance

Water intake (WI) was assessed daily. The water was weighed before being supplied in buckets and weighed again 24 h later. Two buckets of water were placed in the shed next to the animal pens to measure daily evaporation. The water balance (WB) was evaluated according to Al-Ramamneh et al. (2012):

1 $$\begin{aligned}& \text{Total water intake (TWI)} \\ &\quad = \text{water intake via drinking fountain (WID; corrected for evaporation)} \\ &\quad+ \text{water intake via food (WIF)}\end{aligned}$$

WIF was calculated from feed intake × feed moisture content.

2 $$\begin{aligned}& \mathrm{T}\mathrm{o}\mathrm{t}\mathrm{a}\mathrm{l}\:\mathrm{w}\mathrm{a}\mathrm{t}\mathrm{e}\mathrm{r}\:\mathrm{e}\mathrm{x}\mathrm{c}\mathrm{r}\mathrm{e}\mathrm{t}\mathrm{i}\mathrm{o}\mathrm{n}\:\left(\mathrm{T}\mathrm{W}\mathrm{E}\right)\\ &\quad=\mathrm{w}\mathrm{a}\mathrm{t}\mathrm{e}\mathrm{r}\:\mathrm{e}\mathrm{x}\mathrm{c}\mathrm{r}\mathrm{e}\mathrm{t}\mathrm{i}\mathrm{o}\mathrm{n}\:\mathrm{v}\mathrm{i}\mathrm{a}\:\mathrm{u}\mathrm{r}\mathrm{i}\mathrm{n}\mathrm{e}\:\left(\mathrm{W}\mathrm{E}\mathrm{U}\right)\:\\ &\quad+\:\mathrm{w}\mathrm{a}\mathrm{t}\mathrm{e}\mathrm{r}\:\mathrm{e}\mathrm{x}\mathrm{c}\mathrm{r}\mathrm{e}\mathrm{t}\mathrm{i}\mathrm{o}\mathrm{n}\:\mathrm{v}\mathrm{i}\mathrm{a}\:\mathrm{f}\mathrm{e}\mathrm{c}\mathrm{e}\mathrm{s}\:\left(\mathrm{W}\mathrm{E}\mathrm{F}\right)\:\end{aligned}$$

WEU was total urine volume collected daily and WEF was fecal output × fecal moisture.

3 $$\:\mathrm{W}\mathrm{a}\mathrm{t}\mathrm{e}\mathrm{r}\:\mathrm{b}\mathrm{a}\mathrm{l}\mathrm{a}\mathrm{n}\mathrm{c}\mathrm{e}\:=\:\mathrm{t}\mathrm{o}\mathrm{t}\mathrm{a}\mathrm{l}\:\mathrm{w}\mathrm{a}\mathrm{t}\mathrm{e}\mathrm{r}\:\mathrm{i}\mathrm{n}\mathrm{t}\mathrm{a}\mathrm{k}\mathrm{e}\:-\:\mathrm{t}\mathrm{o}\mathrm{t}\mathrm{a}\mathrm{l}\:\mathrm{w}\mathrm{a}\mathrm{t}\mathrm{e}\mathrm{r}\:\mathrm{e}\mathrm{x}\mathrm{c}\mathrm{r}\mathrm{e}\mathrm{t}\mathrm{i}\mathrm{o}\mathrm{n}$$

### Nitrogen balance

Urine was collected once a day in plastic buckets containing 100 mL of 20% hydrochloric acid to prevent nitrogen volatilization and sampled (10% aliquot of total urine) for the determination of nitrogen content. Nitrogen balance (NB) was calculated as described by Silva and Leão (1979):

4 $$\:\mathrm{N}\mathrm{i}\mathrm{t}\mathrm{r}\mathrm{o}\mathrm{g}\mathrm{e}\mathrm{n}\:\mathrm{a}\mathrm{b}\mathrm{s}\mathrm{o}\mathrm{r}\mathrm{b}\mathrm{e}\mathrm{d}\:\left(\mathrm{N}\mathrm{A}\right)\:=\:\mathrm{N}\mathrm{i}\mathrm{t}\mathrm{r}\mathrm{o}\mathrm{g}\mathrm{e}\mathrm{n}\:\mathrm{i}\mathrm{n}\mathrm{g}\mathrm{e}\mathrm{s}\mathrm{t}\mathrm{e}\mathrm{d}\:\left(\mathrm{N}\mathrm{I}\right)\:-\mathrm{N}\mathrm{i}\mathrm{t}\mathrm{r}\mathrm{o}\mathrm{g}\mathrm{e}\mathrm{n}\:\mathrm{f}\mathrm{e}\mathrm{c}\mathrm{e}\mathrm{s}\:\left(\mathrm{N}\mathrm{F}\right)$$

5 $$\:\mathrm{N}\mathrm{i}\mathrm{t}\mathrm{r}\mathrm{o}\mathrm{g}\mathrm{e}\mathrm{n}\:\mathrm{r}\mathrm{e}\mathrm{t}\mathrm{a}\mathrm{i}\mathrm{n}\mathrm{e}\mathrm{d}\:\left(\mathrm{N}\mathrm{R}\right)\:=\:\mathrm{N}\mathrm{i}\mathrm{t}\mathrm{r}\mathrm{o}\mathrm{g}\mathrm{e}\mathrm{n}\:\mathrm{a}\mathrm{b}\mathrm{s}\mathrm{o}\mathrm{r}\mathrm{b}\mathrm{e}\mathrm{d}\:-\:\mathrm{N}\mathrm{i}\mathrm{t}\mathrm{r}\mathrm{o}\mathrm{g}\mathrm{e}\mathrm{n}\:\mathrm{u}\mathrm{r}\mathrm{i}\mathrm{n}\mathrm{e}\:\left(\mathrm{N}\mathrm{U}\right)$$

6 $$\:\mathrm{N}\mathrm{i}\mathrm{t}\mathrm{r}\mathrm{o}\mathrm{g}\mathrm{e}\mathrm{n}\:\mathrm{B}\mathrm{a}\mathrm{l}\mathrm{a}\mathrm{n}\mathrm{c}\mathrm{e}\:=\:(\mathrm{N}\mathrm{i}\mathrm{t}\mathrm{r}\mathrm{o}\mathrm{g}\mathrm{e}\mathrm{n}\:\mathrm{r}\mathrm{e}\mathrm{t}\mathrm{a}\mathrm{i}\mathrm{n}\mathrm{e}\mathrm{d}\:/\:\mathrm{N}\mathrm{i}\mathrm{t}\mathrm{r}\mathrm{o}\mathrm{g}\mathrm{e}\mathrm{n}\:\mathrm{i}\mathrm{n}\mathrm{g}\mathrm{e}\mathrm{s}\mathrm{t}\mathrm{e}\mathrm{d})\:\mathrm{*}100$$

Nitrogen intake was calculated from feed intake and dietary nitrogen concentration. Fecal and urinary nitrogen were determined from total fecal and urine output and their respective nitrogen concentrations.

### Animal performance

Animals were weighed at the beginning and end of the experimental period, after 16 h of solid fasting (with access to water) to obtain initial body weight (IBW) and final body weight (FBW). Average daily gain (ADG) and feed efficiency (FE) were determined according to the equations below:

7 $$\:ADG=\frac{FBW-IBW}{\mathrm{d}\mathrm{a}\mathrm{y}\mathrm{s}\:\mathrm{o}\mathrm{f}\:\mathrm{c}\mathrm{o}\mathrm{n}\mathrm{f}\mathrm{i}\mathrm{n}\mathrm{e}\mathrm{m}\mathrm{e}\mathrm{n}\mathrm{t}}$$

8 $$\:FE=\:\frac{Total\:weight\:gain}{DMI}$$

### Carcass yield

At the end of the experimental period, lambs were slaughtered. Animals were previously stunned and immediately slaughtered by bleeding by cutting the carotid arteries and jugular veins (Brasil 2017). Subsequently, skinning, evisceration, and removal of the head (sectioned at the atlanto-occipital joint) and extremities of the limbs (sectioned at the metacarpal and metatarsal joints) were performed. Carcasses were identified, washed, and weighed with kidneys and kidney fat to obtain hot carcass weight (HCW) and hot carcass yield (HCY). Carcasses were transferred to a cold room (4 °C; 24 h) and hung by the tendons on appropriate hooks. Then, carcasses were weighed to obtain the cold carcass weight (CCW) and cold carcass yield (CCY), and the determination of cooling losses (CL). The equations described by Cartaxo et al. (2009) were used:

9 $$\:\mathrm{H}\mathrm{C}\mathrm{Y}\:=\:\frac{HCW}{\mathrm{s}\mathrm{l}\mathrm{a}\mathrm{u}\mathrm{g}\mathrm{h}\mathrm{t}\mathrm{e}\mathrm{r}\:\mathrm{b}\mathrm{o}\mathrm{d}\mathrm{y}\:\mathrm{w}\mathrm{e}\mathrm{i}\mathrm{g}\mathrm{h}\mathrm{t}}\mathrm{*}100\:$$

10 $$\:\mathrm{C}\mathrm{C}\mathrm{Y}=\:\frac{CCW}{\mathrm{s}\mathrm{l}\mathrm{a}\mathrm{u}\mathrm{g}\mathrm{h}\mathrm{t}\mathrm{e}\mathrm{r}\:\mathrm{b}\mathrm{o}\mathrm{d}\mathrm{y}\:\mathrm{w}\mathrm{e}\mathrm{i}\mathrm{g}\mathrm{h}\mathrm{t}}*100$$

11 $$\:CL=\:\frac{(HCW-CCW)}{HCW}*100$$

### Chemical analysis

Samples of the ingredients, diets, leftovers, and feces were pre-dried in a forced-air oven at 55 °C for 72 h and ground to 1-mm particles (Wiley Mill, Marconi, MA-580, Piracicaba, Brazil). Chemical analyses were performed using the procedures described by the Association of Analytical Chemists (AOAC 2016) for dry matter (DM), mineral matter (MM), crude protein (CP), ether extract (EE), and acid detergent fiber (ADF), using the protocols 967.03; 942.05; 981.10; 920.29; and 973.18, respectively. Neutral detergent fiber (NDF) (Van Soest et al. 1991), and neutral detergent fiber corrected for ash and protein (NDFap) were determined according to Mertens (2002). Lignin in sulfuric acid (LIG) was determined as proposed by Silva and Queiroz (2002). Neutral detergent insoluble nitrogen (NDIN), acid detergent insoluble nitrogen (ADIN), neutral detergent insoluble protein (NDIP), and acid detergent insoluble protein (ADIP) were determined according to Detmann et al. (2010), and starch was determined by Queiroz et al. (2008).

Hemicellulose (HEM), cellulose (CEL), total carbohydrates (TC; Sniffen et al. (1992), non-fiber carbohydrates (NFC; Hall 2003), and total digestible nutrients (TDN; Harlan et al. 1991) were estimated by the equations:

12 $$\mathrm{HEM} = \mathrm{NDF} - \mathrm{ADF}$$

13 $$\mathrm{CEL} = \mathrm{ADF} - \mathrm{LIG}$$

14 $$\mathrm{TC} = 100 - (\mathrm{CP} + \mathrm{EE} + \mathrm{MM})$$

15 $$\mathrm{NFC} = \mathrm{TC} - \mathrm{NDFap}$$

16 $$\mathrm{TDN} = 82.75 - (0.704 \times \mathrm{ADF})$$

The apparent digestibility coefficient of the nutrients was calculated as described by Silva and Leão (1979):

17 $$\:ADC=\:\frac{(\mathrm{N}\mathrm{u}\mathrm{t}\mathrm{r}\mathrm{i}\mathrm{e}\mathrm{n}\mathrm{t}\mathrm{s}\:\mathrm{i}\mathrm{n}\mathrm{g}\mathrm{e}\mathrm{s}\mathrm{t}\mathrm{e}\mathrm{d}\:-\:\mathrm{N}\mathrm{u}\mathrm{t}\mathrm{r}\mathrm{i}\mathrm{e}\mathrm{n}\mathrm{t}\mathrm{s}\:\mathrm{e}\mathrm{x}\mathrm{c}\mathrm{r}\mathrm{e}\mathrm{t}\mathrm{e}\mathrm{d})}{\mathrm{N}\mathrm{u}\mathrm{t}\mathrm{r}\mathrm{i}\mathrm{e}\mathrm{n}\mathrm{t}\mathrm{s}\:\mathrm{i}\mathrm{n}\mathrm{g}\mathrm{e}\mathrm{s}\mathrm{t}\mathrm{e}\mathrm{d}}*100$$

### Statistical analysis

Data were analyzed using a randomized complete block design with a 2 × 2 factorial arrangement with the GLM procedure in SAS (version 9.4; SAS Institute Inc., Cary, NC, USA). The fixed effects included the presence or absence of corn cobs, the inclusion or absence of butterfly pea hay, and their interaction, while initial body weight was used as the blocking factor. When significant interactions were detected, means were compared using Tukey’s test at a 5% probability level. The statistical model used was:

18 $$\:{Y}_{\left\{ijkl\right\}}\:=\mu\:\:+\:{B}_{i}\:+\:{C}_{j}\:+\:{H}_{k}\:+\:{\left(C*\:H\right)}_{jk}\:+{\epsilon\:}_{ijkl}\:$$

where $\:{Y}_{\left\{ijkl\right\}}$ is the observed response variable; $\:\mu\:$ is the overall mean; $\:{B}_{i}$ is the effect of the i-th block based on initial body weight; $\:{C}_{j}$ is the fixed effect of corn cob presence or absence; $\:{H}_{k}$ is the fixed effect of butterfly pea hay inclusion or absence; $\:{\left(C*\:H\right)}_{jk}$ is the interaction effect between corn cob presence and butterfly pea hay inclusion; and $\:{\epsilon\:}_{ijkl}\:$ is the random residual error associated with each observation.

## Results

### Intake, digestibility, and animal performance

EE intake was influenced by the interaction between butterfly pea hay (BH) inclusion and the presence of corn cobs (*P* = 0.005). Lambs fed CSC and CSWC diets presented lower EE intake than those receiving CSC + BH and CSWC + BH (Table 4). The inclusion of BH in corn silages increased dry matter (DM; *P* < 0.001), organic matter (OM; *P* < 0.001), CP (*P* < 0.001), total carbohydrates (TC; *P* < 0.001), and non-fiber carbohydrates (NFC; *P* < 0.001) intake. NDF intake was not affected by BH inclusion (*P* = 0.517), and the corn cobs factor did not influence DM or nutrient intake variables (*P* > 0.05; Table 4).

**Table 4:** Intake of dry matter and nutrients, apparent digestibility of nutrients, fecal starch and performance of lambs fed diets containing different silages including ensiled corn with cobs + butterfly pea hay (CSC + BH); ensiled corn without cobs + butterfly pea hay (CSWC + BH), ensiled corn with cobs without butterfly pea hay (CSC); ensiled corn without cobs without butterfly pea hay (CSWC)

Items	Silages			SEM	P-Value			
	CSC + BH	CSWC + BH	CSC	CSWC		BH	C	BH × C
	Intake (g/day)							
DM	1136.90	1050.96	787.73	802.47	46.80	<0.001	0.463	0.305
OM	1054.25	974.76	741.26	751.83	42.76	<0.001	0.447	0.325
NDF	442.28	396.00	409.51	399.36	14.66	0.517	0.224	0.428
CP	169.62	162.06	118.47	123.66	7.03	<0.001	0.871	0.394
EE	29.81	23.43	21.10	22.96	1.10	0.002	0.091	0.005
TC	855.03	789.06	598.45	608.49	34.77	<0.001	0.446	0.305
NFC	412.74	395.96	188.93	209.13	25.58	<0.001	0.909	0.234
	Digestibility (%)							
DM	63.01	60.31	64.69	63.43	0.47	0.001	0.005	0.245
CP	82.07	80.81	79.52	74.40	0.69	<0.001	<0.001	0.006
NDF	60.40	57.46	48.71	44.14	1.85	0.002	0.130	0.731
FS	88.50	80.84	73.51	46.74	4.52	0.001	0.015	0.144
	Performance							
FBW	34.20	32.50	30.95	30.12	2.30	0.043	0.125	0.555
ADG	172.40	155.20	138.40	128.60	8.07	0.062	0.379	0.806
FE	0.151	0.147	0.176	0.162	0.05	<0.001	0.395	0.212

DM: Dry matter; OM: Organic matter; NDF: Neutral detergent fiber; CP: Crude protein; EE: ether extract; TC: Total carbohydrates; NFC: non-fibrous carbohydrates; FS: Fecal starch; FBW: Final body weight; ADG: Average daily gain; FE: Feed efficiency; SEM: Standard error of the mean; P value: Probability value; BH: Butterfly pea hay; C: Cobs; BH x C = Interaction effect between butterfly pea hay x cobs; df: residual degrees of freedom = 12

Regarding nutrient digestibility, CP digestibility was affected by the interaction between BH and corn cobs (*P* = 0.006), with lower values observed in lambs fed CSC and CSWC compared with those receiving CSC + BH and CSWC + BH (Table 4). No interaction effects were detected for the remaining digestibility variables. Nevertheless, independent effects of BH and corn cobs were observed for dry matter (DM) digestibility (*P* < 0.05), with reduced DM digestibility particularly in lambs fed CSWC + BH. NDF digestibility was influenced only by BH inclusion (*P* = 0.002), with lower values recorded in lambs fed diets without BH (CSC and CSWC), while the corn cobs factor had no effect (*P* = 0.130). For fecal starch (FS), both BH (*P* = 0.001) and corn cobs (*P* = 0.015) showed isolated effects, with diets containing corn without cobs resulting in lower FS digestibility compared with diets containing corn with cobs (Table 4).

Animal performance responses were primarily associated with BH inclusion. Lambs receiving CSC + BH and CSWC + BH showed higher final body weight (FBW) (*P* = 0.043). In contrast, greater feed efficiency (FE) values were observed in lambs fed silages without BH (CSC and CSWC) (*P* < 0.001). No interaction between BH and corn cobs was detected for final body weight (FBW), average daily gain (ADG), or feed efficiency (FE) (*P* > 0.05 for interaction effects). The corn cobs factor also did not affect FBW (*P* = 0.125), ADG (*P* = 0.379), or FE (*P* = 0.395). Butterfly pea hay inclusion increased FBW (*P* = 0.043) and reduced FE (*P* < 0.001), whereas ADG was not significantly affected (*P* = 0.062). (Table 4).

### Water balance and nitrogen balance

Water intake via drinking fountain (WID) was affected by the interaction between butterfly pea hay (BH) inclusion and the presence of corn cobs (*P* = 0.002). Lambs fed CSC + BH and CSWC + BH showed greater WID compared with those receiving CSC and CSWC diets (Table 5). In contrast, water intake via feed (WIF) was influenced only by BH inclusion (*P* < 0.001), with higher values observed in lambs fed silages without BH (CSC and CSWC). Total water intake (TWI; *P* = 0.179), water excretion via urine (WEU; *P* = 0.668), total water excretion (TWE; *P* = 0.216), retained water (RW; *P* = 0.353), and water balance (WB; *P* = 0.448) were not affected by BH inclusion (Table 5). Although no interaction between factors was detected for water excretion via feces (WEF), isolated effects of both BH (*P* = 0.006) and corn cobs (*P* = 0.047) were observed, with lower WEF values recorded in lambs fed CSC compared with those receiving CSWC + BH. The corn cobs factor also influenced WEU (*P* = 0.039), TWE (*P* = 0.019), and WB (*P* = 0.021). Lambs fed diets containing corn with cobs (CSC + BH and CSC) showed lower total water excretion and greater water balance compared with lambs fed diets without cobs (Table 5). No isolated effect of the cobs factor was observed for WIF (*P* = 0.149), WID (*P* = 0.267), TWI (*P* = 0.110), or RW (*P* = 0.850) (Table 5).

**Table 5:** Water balance and nitrogen balance of lambs fed diets containing different silages including ensiled corn with cobs + butterfly pea hay (CSC + BH); ensiled corn without cobs + butterfly pea hay (CSWC + BH), ensiled corn with cobs without butterfly pea hay (CSC); ensiled corn without cobs without butterfly pea hay (CSWC)

Items	Silages				SEM	*P*-Value		
	CSC + BH	CSWC + BH	CSC	CSWC		BH	C	BH × C
	Water balance (kg/day)							
WIF	0.990	1.144	2.232	2.502	0.16	< 0.001	0.149	0.681
WID	1.880	2.288	0.646	0.420	0.18	< 0.001	0.267	0.002
TWI	2.868	3.428	2.880	2.920	0.09	0.179	0.110	0.161
WEF	0.522	0.644	0.352	0.418	0.03	0.006	0.047	0.523
WEU	0.518	0.876	0.692	0.788	0.05	0.668	0.039	0.206
TWE	1.036	1.520	1.044	1.202	0.07	0.216	0.019	0.195
RW	1.828	1.908	1.836	1.720	0.05	0.353	0.850	0.314
WB, %	63.81	55.79	63.93	59.38	1.28	0.448	0.021	0.477
	Nitrogen balance (g/day)							
NI	25.50	26.80	19.50	19.77	0.96	< 0.001	0.488	0.649
NF	6.61	8.82	4.55	4.99	0.42	< 0.001	0.002	0.024
NU	9.64	10.23	7.12	8.25	0.58	0.045	0.412	0.792
NA	18.89	17.98	14.95	14.78	0.60	0.006	0.502	0.641
NR	9.25	7.75	7.83	6.53	0.63	0.343	0.317	0.942
NB, %	36.79	28.88	41.17	29.48	2.72	0.666	0.107	0.743

WIF: Water intake via food; WID: Water intake via drinking fountain; TWI: Total water intake; WEF: Water excretion via feces; WEU: Water excretion via urine; TWE: total water excretion; RW: Retained water; WB: Water balance; NI: Nitrogen ingested; NF: Nitrogen feces; NU: Nitrogen urine; NA: Nitrogen absorbed; NR: Nitrogen retained; NB: Nitrogen balance; SEM: Standard error of the mean; P value: Probability value; BH: Butterfly pea hay; C: Cobs; BH x C = Interaction effect between butterfly pea hay × cobs; df: residual degrees of freedom = 12

Fecal nitrogen excretion (NF) was influenced by the interaction between BH inclusion and corn cobs (*P* = 0.024), with higher NF values observed in lambs fed CSC + BH and CSWC + BH compared with those receiving CSC and CSWC diets (Table 5). No interaction effects were detected for nitrogen intake (NI; *P* = 0.649), urinary nitrogen excretion (NU; *P* = 0.792), nitrogen absorbed (NA; *P* = 0.641), nitrogen retained (NR; *P* = 0.942), or nitrogen balance (NB; *P* = 0.743). Independent effects of BH inclusion were observed for NI (*P* < 0.001), NU (*P* = 0.045), and NA (*P* = 0.006). Lambs receiving CSC + BH and CSWC + BH showed higher nitrogen intake, urinary nitrogen excretion, and nitrogen absorption compared with lambs fed corn silages without BH. However, BH inclusion did not affect NR (*P* = 0.343) or NB (*P* = 0.666) (Table 5). The corn cobs factor did not influence NI (*P* = 0.488), NU (*P* = 0.412), NA (*P* = 0.502), NR (*P* = 0.317), or NB (*P* = 0.107) (Table 5).

### Carcass yield

Carcass weights, carcass yields, and cooling losses were not affected by the interaction between butterfly pea hay (BH) inclusion and the presence of corn cobs (*P* > 0.05; Table 6). An isolated effect of BH was observed for hot carcass weight (HCW) (*P* = 0.011) and cold carcass weight (CCW) (*P* = 0.009), with greater carcass weights observed in lambs fed diets containing BH. However, BH inclusion did not affect hot carcass yield (HCY; *P* = 0.383), cold carcass yield (CCY; *P* = 0.212), or cooling losses (CL; *P* = 0.133) (Table 6).

**Table 6:** Weights and carcass yields of lambs fed diets containing different silages including ensiled corn with cobs + butterfly pea hay (CSC + BH); ensiled corn without cobs + butterfly pea hay (CSWC + BH), ensiled corn with cobs without butterfly pea hay (CSC); ensiled corn without cobs without butterfly pea hay (CSWC)

Items	Silages				SEM	*P*-Value		
	CSC + BH	CSWC + BH	CSC	CSWC		BH	C	BH × C
HCW, kg	14.66	13.80	13.24	12.37	0.43	0.011	0.095	0.987
CCW, kg	14.24	13.44	12.82	12.00	0.42	0.009	0.106	0.979
HCY, %	45.90	44.10	45.50	43.03	0.45	0.383	0.021	0.681
CCY, %	44.30	43.88	42.64	41.76	0.63	0.212	0.656	0.873
CL, %	2.84	2.63	3.19	2.96	0.11	0.133	0.316	0.963

HCW: Hot carcass weight; CCW: Cold carcass weight; HCY: Hot carcass yield; CCY: Cold carcass yield; CL: Cooling loss; SEM: Standard error of the mean; P value: Probability value; BH: Butterfly pea hay; C: Cobs; BH x C = Interaction effect between butterfly pea hay x cobs; df: residual degrees of freedom = 12

The corn cobs factor affected only hot carcass yield (HCY) (*P* = 0.021), with lambs fed diets without cobs (CSWC) showing lower HCY than those fed diets containing corn with cobs. No isolated effect of the cobs factor was detected for HCW (*P* = 0.095), CCW (*P* = 0.106), CCY (*P* = 0.656), or CL (*P* = 0.316) (Table 6).

## Discussion

The inclusion of butterfly pea hay (BH) improved the chemical composition of the mixed silages, likely contributing to greater animal acceptance and reflected in increased dry matter (DM) and nutrient intake. The higher intake observed in lambs fed diets containing BH may be associated with the DM and fiber characteristics of these silages. According to Hawu et al. (2022), the inclusion of legume hay in corn ensiling can reduce NDF content through hemicellulose hydrolysis, increasing the availability of soluble carbohydrates for lactic acid production during fermentation. The low pH values observed in the silages may be associated with the high moisture content of the corn material, elevated compaction density, and the efficient anaerobic conditions achieved in the small hermetically sealed silage bags. These conditions likely favored rapid lactic acid fermentation and accelerated acidification during ensiling.

In diets containing CSC and CSWC without BH, cottonseed meal was included as a protein source, increasing the NDF content of these diets and possibly contributing to reduced DM intake. Further studies evaluating the nutritional composition of total mixed rations containing mixed corn silages, with or without cobs, and associated with BH and alternative protein sources may contribute to a better understanding of intake responses in small ruminants.

The inclusion of BH in corn silages resulted in greater NDF digestibility, which provides rapid rumen emptying, due to the increase in the digesta passage rate, and provides an increase in DM and nutrient intake, and fecal starch (Pereira et al. 2018), which favored the results obtained, not interfering with animal performance, which presented daily gains above 150 g established during the formulation diets.

The lower DM digestibility observed in lambs that received diets based on CSWC + BH may be related to the ADF and lignin levels in the ingredients of this diet. The absence of cobs reduced grain content in the silage and, consequently, starch, which is highly rumen-degradable, reducing DM digestibility. Silva et al. (2021a, b) reported that the DM digestibility of corn silages produced in Brazil is 59.6%; therefore, the DM digestibility of the silages in the present study is above this value, suggesting high nutrient utilization, regardless of the addition of BH to the silages.

Lambs on diets containing corn silages with BH showed higher CP digestibility. The relatively high lignin concentration observed in butterfly pea hay may be related to the maturity of the plants at harvest, which occurred 110 days after planting during the flowering stage, as well as to the inclusion of branches and pods in the harvested biomass. In addition, differences in lignin determination methodology may contribute to variation among values reported for C. ternatea in the literature. Although these silages have a higher lignin content, which can bind to protein and limit the action of microorganisms during digestion (Silva et al. 2023), this was not sufficient to negatively affect CP digestibility. Araújo et al. (2022) tested diets containing varying levels of butterfly pea hay and cactus pear in Boer goats and reported CP digestibility ranging from 71.52 to 76.39%, lower than the values observed in the present study with the inclusion of BH in corn silages.

The greater FS in animals that received diets containing CSC + BH and CSWC + BH can be explained by the characteristics of the forages used to make the silages. Diets containing CSC benefited from the presence of grains and from the timing of corn harvest (just before 2/3 of the milk line, which is recommended for silages).

BH also contributed to the digestibility of starch in silages, possibly due to starch present in seeds within their pods, since the entire aerial part of the plant was used at the time of haying. Thus, increasing the amount of starch in the diet increased starch digestion in the rumen, resulting in greater fecal starch output. In addition, diets containing CSC + BH and CSWC + BH had lower NDF content, which led to higher DM intake and possibly increased the passage rate, increasing starch escape from the rumen.

The lower feed efficiency observed in lambs fed BH-containing silages resulted from a greater increase in dry matter intake than in average daily gain. Thus, although BH inclusion increased nutrient intake and improved final body weight and carcass weights, the increase in weight gain was not proportional to the additional feed consumed. Similar responses have been reported for diets containing highly palatable legume forages or supplements that stimulate intake without a proportional improvement in animal performance (Van Soest 1994; NRC 2007).

Higher DM intake in animals fed BH-containing silages was accompanied by greater water intake via drinking fountain, reflecting compensatory mechanisms associated with lower water intake from feed and the need to facilitate bolus formation and digesta transport through the gastrointestinal tract (Santos et al. 2019; Silva et al. 2021a, b). Increased urinary and total water excretion in animals fed diets without cobs may be associated with higher total water intake, as described by Thiet et al. (2022), who reported that increased water consumption leads to proportional increases in renal excretion to maintain osmotic balance.

The greater number of cobs in CSC and CSC + BH, and consequently the carbohydrates, influenced the WB of the animals, as they contributed to ruminal physiological processes and thus optimized the use of nutrients that have water in their composition. It is noteworthy that the study did not consider losses inherent to perspiration, which represent about 70% of total losses, according to Souza et al. (2013).

Higher fecal nitrogen excretion in animals fed diets containing BH is consistent with the greater nitrogen intake and absorption observed in these treatments. As nitrogen intake exceeded animal requirements (19.7 g animal^− 1^ day^− 1^; NRC 2007), excess nitrogen was excreted via feces and urine (Geron et al. 2018). Mora-Luna et al. (2022) reported that fecal nitrogen excretion increases with fermentation activity in the large intestine, particularly in diets rich in grains such as corn.

The lower hot carcass weight observed in lambs fed CSWC diets may be associated with the reduced crude protein intake and lower dry matter digestibility observed in these animals. Diets containing butterfly pea hay promoted greater nutrient intake and improved crude protein digestibility, which likely contributed to greater carcass weight. However, average daily gain was not significantly affected by the treatments, indicating that carcass responses were more closely associated with nutrient utilization than with differences in growth rate. No significant effects were observed for cold carcass yield or cooling losses. Additional studies evaluating non-carcass components and meat quality characteristics may improve understanding of the effects of mixed silages on carcass composition in lambs.

Some limitations of the present study should be considered when interpreting the results. The relatively small number of experimental units per treatment and the variability in initial body weight among the lambs may have reduced the sensitivity to detect differences between treatments in certain productive variables, particularly average daily gain. The absence of measurements of ruminal fermentation also limits a more mechanistic interpretation of the observed effects on nitrogen utilization. In addition, although the 5-day adaptation period to the metabolic cages was considered adequate for the experimental protocol, its relatively short duration may have contributed to some residual variability in digestibility and water and nitrogen balance measurements. Therefore, additional studies incorporating larger experimental populations and assessments of ruminal fermentation are recommended.

## Conclusion

Under the experimental conditions of this study, the inclusion of 30% butterfly pea hay in corn silage-based diets increased dry matter and crude protein intake and improved crude protein digestibility and carcass weight of lambs. The maintenance of corn cobs in the silage improved starch digestibility, water balance, and hot carcass yield, indicating that cob removal may reduce the energetic value of the diet. Although butterfly pea hay inclusion did not significantly affect average daily gain, it reduced feed efficiency because the increase in dry matter intake was proportionally greater than the increase in body weight gain. Nevertheless, BH inclusion increased final body weight and carcass weights and partially mitigated the nutritional limitations of corn silages without cobs by sustaining greater nutrient intake and utilization. Therefore, butterfly pea hay may be used as a forage additive in corn silage-based diets for lambs.

## Data Availability

No applicable.

## Code availability

No applicable.
